# Non-Coding RNA in Penile Cancer

**DOI:** 10.3389/fonc.2022.812008

**Published:** 2022-05-13

**Authors:** Jaqueline Diniz Pinho, Gyl Eanes Barros Silva, Antonio Augusto Lima Teixeira-Júnior, Thalita Moura Silva Rocha, Lecildo Lira Batista, Amanda Marques de Sousa, José de Ribamar Rodrigues Calixto, Rommel Rodrigues Burbano, Carolina Rosal Teixeira de Souza, André Salim Khayat

**Affiliations:** ^1^ Zé Doca Center for Higher Studies, State University of Maranhão, Zé Doca, Brazil; ^2^ Laboratory of Immunofluorescence and Electron Microscopy, University Hospital of the Federal University of Maranhão, São Luís, Brazil; ^3^ Oncology Research Center, João de Barros Barreto University Hospital, Federal University of Pará, Belém, Brazil; ^4^ Department of Genetics, University of Sao Paulo, Ribeirão Preto, São Paulo, Brazil; ^5^ Coordination of Medicine, Federal University of Amapá, Macapá, Brazil; ^6^ Department of Medicine, University Hospital of the Federal University of Maranhão, São Luís, Brazil; ^7^ Laboratory Biology Molecular, Ophir Loyola Hospital Ophir, Belém, Brazil; ^8^ Institute of Biological Sciences, Federal University of Pará, Belém, Brazil

**Keywords:** non coding RNAs (ncRNAs), penile cancer, biomarkers, piRNAs, miRNA

## Abstract

Penile cancer (PC) still presents a health threat for developing countries, in particular Brazil. Despite this, little progress has been made on the study of markers, including molecular ones, that can aid in the correct management of the patient, especially concerning lymphadenectomy. As in other neoplasms, non-coding RNAs (ncRNAs) have been investigated for penile cancer, with emphasis on microRNAs, piRNAs (PIWI-interacting small RNAs), and long non-coding RNAs (LncRNAs). In this context, this review aims to assemble the available knowledge on non-coding RNA linked in PC, contributing to our understanding of the penile carcinogenesis process and addressing their clinical relevance. ncRNAs are part of the novel generation of biomarkers, with high potential for diagnosis and prognosis, orientating the type of treatment. Furthermore, its versatility regarding the use of paraffin samples makes it possible to carry out retrospective studies.

## Introduction

Penile cancer (PC) is highly incident in developing regions such as Asia, Africa, and South America, with Brazil having the highest incidence rate in the world, 6.15/100,000 inhabitants (1, 2). The etiology of penile cancer is not fully understood, but some risk factors have been strongly associated with this malignant neoplasm. Among them stand out the presence of phimosis, poor hygiene of the organ, and infection by the Human Papilloma Virus (HPV) (1).

HPV prevalence in male genital cancer is highly variable, reflecting differences in sensitivity in the methods used to detect the virus, and also associated with the histological subtype of the tumor, being more frequent in condylomatous and basaloid tumors (3, 4). The global prevalence is 36-40%, with a more significant contribution from subtypes HPV16 and HPV18 (3, 5).

Penectomy is still the “gold standard” for the treatment of primary tumors. It can be partial or total, depending on the extension of the lesion (6, 7). In some patients, lymphadenectomy is essential for surgical management, although it presents risks of complications and has high morbidity. At some health services, this type of procedure has been performed prophylactically, especially in developing regions, where many patients have difficulties maintaining medical care (8). Furthermore, patients without palpable lymph nodes at diagnosis may present micrometastases. The rate of occurrence of micrometastases is 25%, and the involvement of more than two inguinal lymph nodes is associated with a greater risk of recurrence (9). Therefore, the concern with lymph node involvement is justified by the significant impact on prognosis (6, 9, 10). Thus, biological markers that can predict or assist in diagnosing this phenomenon are of great clinical importance. Some markers based on ncRNAs have been investigated, especially those associated with lymph node metastasis (11, 12), perineural invasion (13), and HPV (14).

**Figure 1:**
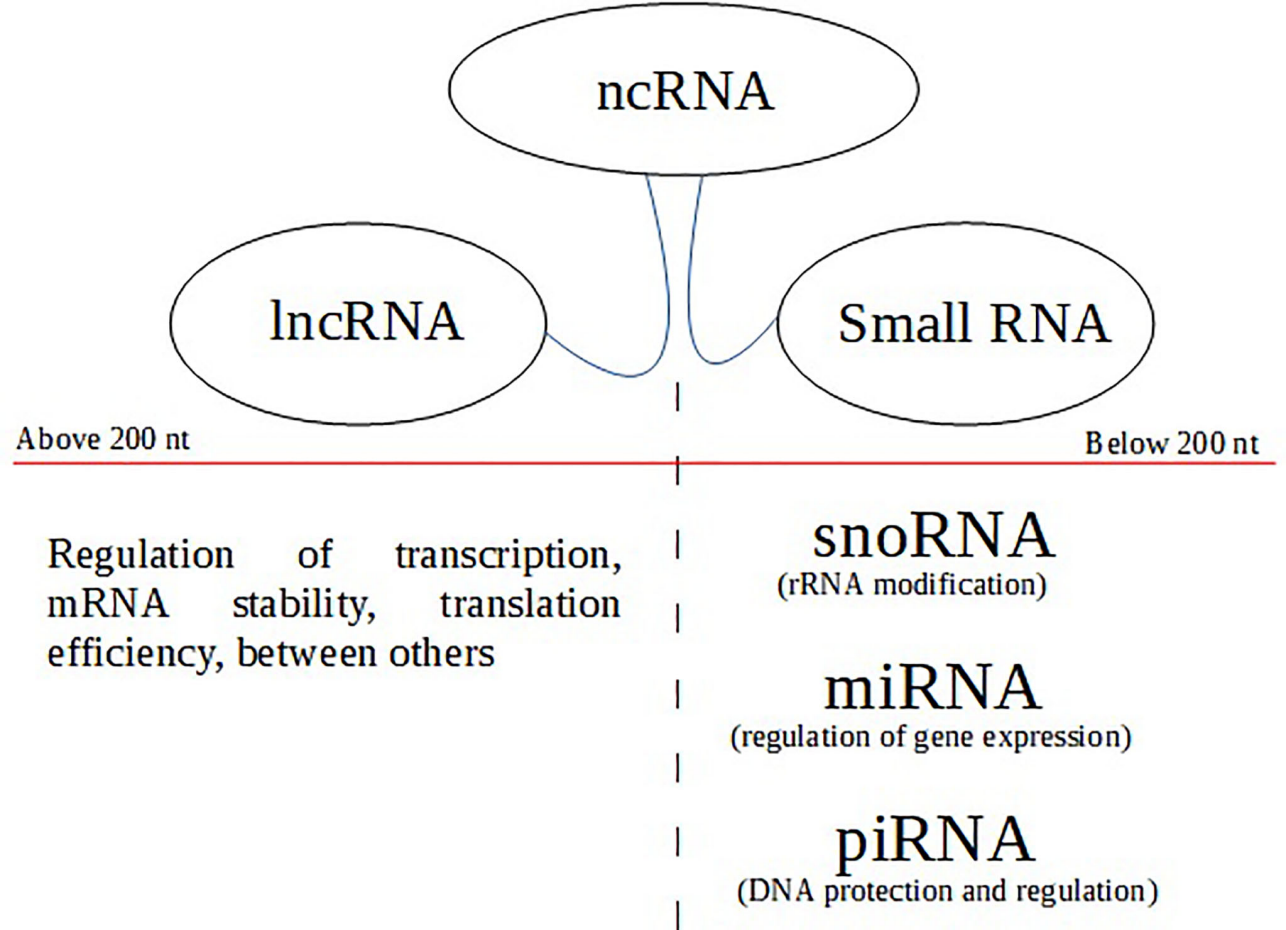
The top of the flowchart (balloons) represents non-coding RNA types based on transcript length (nt). Below the nt size divider (red line) are the subclass with their respective functions.

For several decades, ncRNAs were considered ‘evolutionary junk.’ They can be classified according to their size, with those up to 200 nucleotides in length being considered small non-coding RNAs (sncRNA). Those with more than 200 nucleotides are long non-coding RNAs (lncRNA). Among the sncRNAs, we highlight microRNAs, piRNAs, and snoRNAs (Small nucleolar RNAs) (15) (**Figure 1**). When interacting with DNA, RNA, or proteins, ncRNAs have many essential functions, such as epigenetic regulation, chromatin remodeling, protein modification, and RNA degradation. Furthermore, they can function as important regulators of gene expression and play crucial roles in many physiological and pathological processes, so much that the abnormal expression of these sncRNAs is involved in many human diseases, including cancer (16).

ncRNAs are involved in the deregulation of several signaling pathways, similar to miRNAs that have several target genes that regulate the expression of epithelial mesenchymal transition (EMT) transcription factors, and also direct genes involved in the encoding of signaling mediators, adhesion junction and polarity complex proteins (17). LncRNAs have also been documented to be involved in the regulation of key factors such as: oxidative stress and inflammation (18). In addition to these, other ncRNAs have also been considered in crucial processes in cancer, among which we have piRNAs that are involved in apoptosis and proliferation (19), and snoRNAs, involved in invasion and metastasis (20). These data point to the importance of studying ncRNAs as potential biomarkers in PC.

In this context, this review aims to interconnect the information produced on non-coding RNAs addressed in PC, relating them to their clinical importance, with perspectives of use as markers that aid in management, in addition to helping to understand the process of carcinogenesis.

### MicroRNAs and Penile Cancer

MicroRNAs (miRNAs or miRs) are small non-coding RNAs (19-23 nt) involved in regulating gene expression at the transcriptional and post-transcriptional levels. These biomolecules constitute one of the most abundant classes of ncRNAs, being widely studied due to their high mRNA silencing potential, regulating relevant processes of gene expression, such as apoptosis, proliferation, and differentiation (21). Gene regulation and expression occur through the complementarity of microRNA and mRNA in the 3’UTR region, with the consequent degradation or repression of target gene transcripts (22).

The dysregulation in the expression of these biomolecules has been related to different pathologies, including cancer (23, 24). There is evidence that the differential expression allows not only the identification of neoplastic tissue but also the different subtypes of malignant lesions, being also helpful in determining the stage and progression of cancer and prognosis and response to treatment (25). Because of this, microRNAs have been considered potential biomarkers for diagnosis, prognosis, and therapy (22, 24, 25).

**Figure 2:**
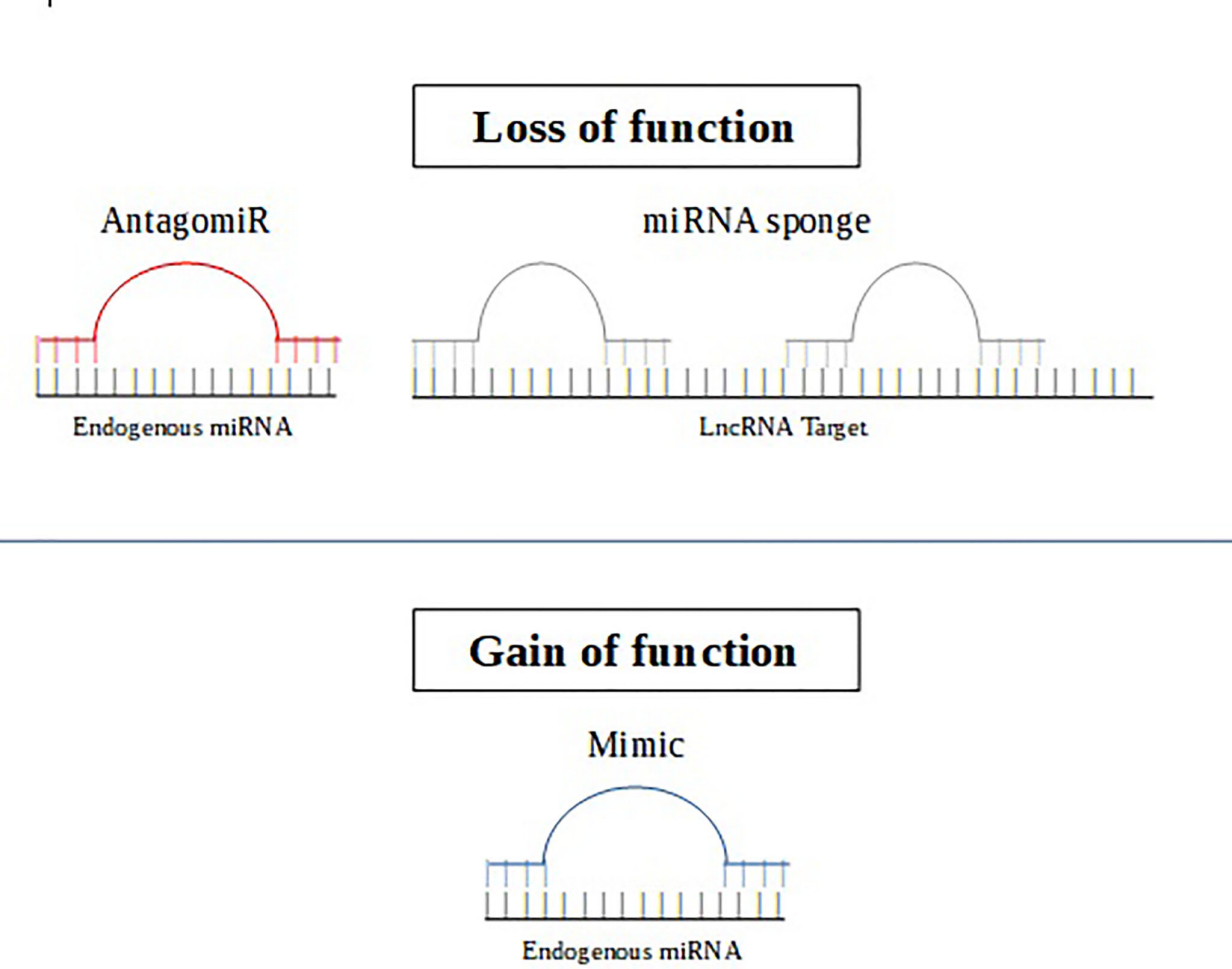
The AntagomiR (in red) is an oligonucleotide sequence complementary to the endogenous target miRNA, leading to functional inhibition. MiRNA sponge (in gray) are RNA transcripts containing binding sites that sequester specific miRNAs to prevent them from interacting with their target sequence. The miRNA mimic (in blue) is an RNA fragment that acts by mimicking an endogenous miRNA that can specifically bind to its target gene.

It is worth noting that there are two forms of therapeutic approaches based on microRNAs. The first approach aims to inhibit the activity of oncogenic miRNAs using miRNA antagonists such as antagomiRs or mimic miRNAs (25). AntagomiRs act by reducing the levels of intracellular overexpression of miRNAs, through their specific binding to mature target miRNA. Meanwhile, mimic miRNAs or mimics are constructed with the aim of replacing the deleted tumor suppressor miRNA (26). The action of antagomiRs and mimics has already been assessed with promising results in malignant neoplasms, such as leukemia (26) and prostate cancer (27). The second specific microRNA therapeutic strategy can be performed using synthetic oligonucleotides that act as microRNA sponges (28) (**Figure 2**).

Furthermore, microRNAs have been used as biomarkers of metastatic disease, which can be termed as metastamiRs. These microRNAs do not influence the initial steps of tumorigenesis, but regulate processes such as transition-mesenchymal epithelium (TEM), apoptosis and angiogenesis (29).

There are few studies that address the role of microRNAs in PC; mainly, they provide important information about HPV infection and/or worse prognostic factors, which are described in **Table 1**.

**Table 1:** Main microRNA linked to penile cancer.

MicroRNAs	Function/Expression	Clinical Significance	Method	References
miR-218	TsmiR/Down	miR-218 was less expressed in hrHPV samples	RT-qPCR	(30)
miR-146a	TsmiR/Down	miR-146a had a decreased expression in hrHPV samples. Its low expression was mediated by oncoprotein E6.	RT-qPCR	(14)
miR-223-3p	oncomiR/Up	Specificity and sensibility to distinct between tumor and non-tumor samples	Microarray/qRT-PCR	(31)
			NGS/qRT-PCR	(32)
		Associated lymph node metastasis.	qRT-PCR	(12)
miR-224-5p	TsmiR/Down	Specificity and sensibility to distinct between tumor and non-tumor samples	Microarray/qRT-PCR	(31)
miR-31-5p	OncomiR/Up	The AR gene is targeted by miR-31-5p. This gene has already been observed as a driver gene in penile cancer.		
miR-145-5p	TsmiR/Down	miR-145-5p targets gene MMP1, which showed increased expression levels in samples from patients with lymph node metastasis	Microarray/qRT-PCR	(31)
		Reduced expression was associated with perineural invasion	qRT-PCR	(13)
miR-1	TsmiR/Down	The reduced expression of these three microRNAs can predict metastasis.	TaqMan Array	(11)
miR-101				
miR-204				
miR-107	OncomiR/Up	High expression when comparing tumor and non-tumor samples.	NGS/qRT-PCR	(32)
		Associated with worsening of prognosis: histological grade II and III, tumors bigger than 2.0 cm, stage III and IV, and lower disease-free survival	qRT-PCR	(12)
miR-21-5p	OncomiR/Up	Was correlated to the absence of PTEN protein expression	qRT-PCR	(12)
miR-137	TsmiR/Down	Reduced expression in patients with lymph node metastasis.	Microarray qRT-PCR	(33)
miR-328-3p				

The first study showcasing the participation of microRNAs in PC was described by Barzon et al. (30). They observed that miR-218 was down-regulated in those samples from patients with high-risk HPV (hrHPV) and with negative protein expression of p53. In oral cancer with HR-HPV+, it has been reported that the dysregulation of miR-218 is mediated by dysregulation oncoprotein E6 (34).

Later, it was also observed that the reduced expression of miR-146a is mediated by oncoprotein E6. The high expression of EGFR (Epidermal Growth Factor Receptor) was associated with the reduced expression (14). The target genes of this microRNA are involved in migration, metastasis formation, and proliferation, such as NOTCH1 (Notch Receptor 1), ROCK1 (Rho Associated Coiled-Coil Containing Protein Kinase 1), and EGFR. The EGFR gene has been extensively studied for PC, and its protein and gene dysregulation has been associated with advanced stage, lower overall survival, and lymph node status. It is, therefore, a vital target marker for therapy (35, 36).

Kuasne et al. (31) found relevant data, who identified some microRNAs with decreased expression (let-7b-5p, miR-185-5p, miR-29b-3p, miR-505-3p miR-146-5p), in a group of seven patients, five of which hrHPV positive. These microRNAs regulate genes; MMP2 (Metalanoprotease 2), MMP9 (Metalanoprotease 9), IGF1R (Insulin Like Growth Factor 1 Receptor), and PTEN (Phosphatase and Tensin homologue), which regulate important mechanisms in the progression of carcinogenesis.In addition to the microRNAs as mentioned above, this same study highlighted three other microRNAs (miR-31-5p, miR-224-5p, and miR-223-3p) that presented high sensitivity and specificity to distinguish between tumoral and non-neoplastic penile tissue. MicroRNA miR-31-5p regulates the AR gene (Androgen Receptor), which is pointed out as the driver gene in penile cancer (37).

Regarding the PTEN gene, it has been reported to be inactivated in several types of cancers (38, 39), including penile cancer (40, 41), either by deletions, mutations, methylation in the promoter region and/or transcriptional post-regulation, through the action of microRNAs (42–44).

As for the relationship between PTEN and microRNAs, it is necessary to mention the data found by Yayu et al. (45), which revealed the increased expression of miR-26a in blood and urine samples from patients with penile cancer. This high expression was associated with low expression of PTEN tumors from HPV-positive patients. The authors suggest that miR-26a can regulate the progression of HPV-positive penile tumors through PTEN modulation.

IGF1R, regulated by the microRNA let-7b-5, is a transmembrane receptor tyrosine kinase that is overexpressed in several malignant neoplasms, including urologic cancers (46). This receptor plays a critical role in cell proliferation, differentiation, and malignant transformation. Protein overexpression of IGF1R has been associated with lower disease-free survival in penile cancer (47).

In PC, the high expression of metalloproteases (MMP2 and MMP9), regulated by miR-29b-3p, correlated with a higher incidence of distant metastasis and lower survival (48).

Only three studies addressed the relationship between alteration in microRNAs expression and lymph node metastasis in PC, as summarized in **Table 1**. Hartz et al. (11) observed that miR-1, miR-101, and miR-204 were under-expressed in penile metastatic tumors. Low expression of miR-1 has been reported for colorectal (49) and cervical (50) cancers. MiR-101 is related to clinical outcomes of worse prognosis in several types of tumors, such as cervical cancer (51) and pancreatic cancer (52), regulating genes such as: mTor (mammalian target of rapamycin), ROCK1, ACKR3 (atypical chemokine receptor 3), MCL1 (MCL1Apoptosis Regulator, BCL2 Family Member) and RAC1 (Rac Family Small GTPase 1), which participate in important pathways in the mechanism of carcinogenesis (53).

In a study carried out by our group, it was possible to identify that the high expression of miR-223-3p is associated with lymph node metastasis. Furthermore, the increased expression of miR-107 and the absence of protein expression of PTEN were observed in patients at more advanced stages of the disease (12). In this same study, we observed that the expression or miR-21 was higher in tumoral samples when compared to non-tumoral ones. According to Gao et al. (54), miR-223-3p can also regulate several pathways in the promotion of tumor metastases, local invasion, transport, extravasation, colonization, and epithelial-mesenchymal transition.

In another study, our group also observed that miR-145-5p is a potential biomarker for perineural invasion (13), an indicator of worse survival in patients with penile cancer (55). MiR-145-5p also has therapeutic potential since the use of mimics of this microRNA in cervical cancer can inhibit cell proliferation (56) and metastasis in ovarian cancer (57).

MiR-21 indirectly modulates PDL-1 expression (58) and miR-145 is able to downregulate the expression of this same marker through its direct binding to 3’UTR (59) PD-L1, which is the main immune checkpoint receptor expressed on cells of the immune system and plays a significant role in cell adhesion, proliferation and cytokine signaling (60). The use of immune checkpoint inhibitors has shown considerable interest as a chemotherapeutic agent in penile cancer and results of clinical trials have provided valuable information for the treatment of aggressive disease (61–63). The use of these two microRNAs can aid in the study and development of these chemotherapeutics, with potential utility in penile cancer, because as we modulate the expression of a microRNA through a single therapeutic approach, the expression of all its target genes returns to baseline.

Recently, Ayoubian et al. (33) identified a low expression of miR-137 and miR-328-3p in usual metastatic penile cancer tumors. Overexpression of miR-137 acts to inhibit tumor growth, in addition to having been assessed as holding therapeutic potential in lung cancer (64). Overexpression of miR-328-3p inhibits cell proliferation, migration, invasion, and transition epithelial-mesenchymal (EMT), acting by inactivating the PI3K/Akt signaling pathway colon-rectal cancer (65).

### piRNAs and Penile Cancer

piRNAs are a type of ncRNA, with a size between 26-31nt. They are so named because they interact with members of the Argonaut family, namely the PIWI (P-element-induced wimpy tests) proteins. With PIWI proteins, piRNAs form a gene silencing complex (66). These silencing complexes act by suppressing transposable elements (TE), which are responsible for maintaining the integrity of the genome, in addition to transcriptionally regulating gene expression, inducing chromatin remodeling and repressing mRNAs that harbor transposon sequences in the 3’UTR or regions 5’UTR (67).

In recent years, some studies have shown, mainly in gastric cancer, that abnormal expression of piRNAs is associated with cancer initiation, progression, and metastasis (67–70). In this context, piRNAs can become a diagnostic tool, therapeutic targets, besides being prognostic cancer biomarkers (67). Using next-generation sequencing, the only work with piRNAs for PC highlighted the ten most abundant piRNAs with a difference in expression when comparing tumor tissue with normal tissue (32). Among the piRNAs highlighted in this work, piR-49145 has already been observed with altered expression in gastric cancer samples compared to adjacent tissue (69).

### Long Non-Coding RNA in Penile Cancer

LncRNAs are transcribed from non-protein-coding mRNAs greater than 200nt. According to their position relative to the protein-coding genes, the lncRNAs can be divided into; a) sense; b) antisense: transcripts located on the opposite strand of protein-coding genes; c) bidirectional; d) intronic: transcripts that are located within introns of protein-coding genes; e) intergenic: lncRNAs that are located in the region between two protein-coding genes (71).

LncRNAs can regulate gene expression through multiple mechanisms, including epigenetic, transcriptional, and post-transcriptional levels. Furthermore, these biomolecules participate in regulating various cellular activities, such as cell differentiation, proliferation, invasion, apoptosis, and autophagy through interaction with RNA, DNA, or proteins (71).

Several studies have shown that LncRNAs are deregulated in pathologies such as cancer, acting as oncogenes or tumor suppressors. Furthermore, these molecules have been identified as clinically useful diagnostic or prognostic biomarkers or therapeutic targets for cancer (71, 72).

In penile cancer, only a single work refers to alterations in LncRNA. Macedo et al. (73) observed amplification in LINC00226 and LINC00221. LINC00221 when positively regulated can serve as a potential diagnostic and prognostic biomarker in hepatocellular cancer (74), and its dysregulation has already been associated with a worse prognosis in cisplatin-resistant non-small cell lung cancer (75), evidencing the relevance of this biomolecule for the carcinogenesis process.

### Perspectives

ncRNAs comprehend the novel generation of biomarkers, with potential use in diagnosis and prognosis, and possibly even aiding in the choice of treatments, especially those with high sensitivity and specificity in distinguishing different tumor stages. The microRNAs discussed in this article are already known to participate in the carcinogenic process. In the literature, some of these have been investigated in clinical routine, using non-invasive samples (blood and urine), such as miR-145-5p and miR-26a, possible targets to be explored in PC. Embora ainda não haja informações sobre o papel destes ncRNAs

In addition, ncRNAs, especially microRNAs, demonstrate to be resistant to the process of formalin-fixed paraffin inclusion, enabling their study in cases where fresh material was not collected and in studies with a retrospective sampling (76). Finally, it is important to consider the importance of researching other ncRNAs such as; snoRNAs, circRNAs (circular RNAs), siRNAs (small interfering RNAs), which have already been observed altered in gastric cancer (44, 69), cervical cancer (51, 56), hepatocellular carcinoma (74) and vulvar cancer (72) in order to understand the role of these biomolecules in penile carcinogenesis.

## Author Contributions

Conception and design: JP, GS, AT, and AK. Administrative support: AT, JP. Provision of study materials or patients: AT, JC, and AK. Collection and assembly of data: JP, AT. Data analysis and interpretation: JP, GS, AK, RB. Manuscript writing: All authors. Final approval of manuscript: All authors.

## Funding

The study was supported by the Fundação de Amparo a Pesquisa (FAPEMA) and Oncology Research Center, João de Barros Barreto University Hospital, Federal University of Pará, Belém, Brazil.

## Conflict of Interest

The authors declare that the research was conducted in the absence of any commercial or financial relationships that could be construed as a potential conflict of interest.

## Publisher’s Note

All claims expressed in this article are solely those of the authors and do not necessarily represent those of their affiliated organizations, or those of the publisher, the editors and the reviewers. Any product that may be evaluated in this article, or claim that may be made by its manufacturer, is not guaranteed or endorsed by the publisher.
